# Does Reduced IGF-1R Signaling in *Igf1r* ^+/−^ Mice Alter Aging?

**DOI:** 10.1371/journal.pone.0026891

**Published:** 2011-11-23

**Authors:** Alex F. Bokov, Neha Garg, Yuji Ikeno, Sachin Thakur, Nicolas Musi, Ralph A. DeFronzo, Ning Zhang, Rebecca C. Erickson, Jon Gelfond, Gene B. Hubbard, Martin L. Adamo, Arlan Richardson

**Affiliations:** 1 Barshop Institute for Longevity and Aging Studies, University of Texas Health Science Center at San Antonio, San Antonio, Texas, United States of America; 2 Department of Cellular and Structural Biology, University of Texas Health Science Center at San Antonio, San Antonio, Texas, United States of America; 3 Department of Biochemistry, University of Texas Health Science Center at San Antonio, San Antonio, Texas, United States of America; 4 Department of Epidemiology and Biostatistics, University of Texas Health Science Center at San Antonio, San Antonio, Texas, United States of America; 5 Department of Pathology, University of Texas Health Science Center at San Antonio, San Antonio, Texas, United States of America; 6 Department of Medicine, University of Texas Health Science Center at San Antonio, San Antonio, Texas, United States of America; 7 Geriatric Research, Education, and Clinical Center (GRECC), South Texas Veterans Health Care System, San Antonio, Texas, United States of America; 8 College of Natural Sciences, University of Texas at Austin, Austin, Texas, United States of America; The University of Hong Kong, Hong Kong

## Abstract

Mutations in insulin/IGF-1 signaling pathway have been shown to lead to increased longevity in various invertebrate models. Therefore, the effect of the haplo- insufficiency of the IGF-1 receptor (*Igf1r^+/−^*) on longevity/aging was evaluated in C57Bl/6 mice using rigorous criteria where lifespan and end-of-life pathology were measured under optimal husbandry conditions using large sample sizes. *Igf1r^+/−^* mice exhibited reductions in IGF-1 receptor levels and the activation of Akt by IGF-1, with no compensatory increases in serum IGF-1 or tissue IGF-1 mRNA levels, indicating that the *Igf1r^+/−^* mice show reduced IGF-1 signaling. Aged male, but not female *Igf1r^+/−^* mice were glucose intolerant, and both genders developed insulin resistance as they aged. Female, but not male *Igf1r^+/−^* mice survived longer than wild type mice after lethal paraquat and diquat exposure, and female *Igf1r^+/−^* mice also exhibited less diquat-induced liver damage. However, no significant difference between the lifespans of the male *Igf1r^+/−^* and wild type mice was observed; and the mean lifespan of the *Igf1r^+/−^* females was increased only slightly (less than 5%) compared to wild type mice. A comprehensive pathological analysis showed no significant difference in end-of-life pathological lesions between the *Igf1r^+/−^* and wild type mice. These data show that the *Igf1r^+/−^* mouse is not a model of increased longevity and delayed aging as predicted by invertebrate models with mutations in the insulin/IGF-1 signaling pathway.

## Introduction

One of the major discoveries in aging during the past decade has been the observation that mutations in insulin/IGF-1 signaling led to increased longevity in various invertebrate models [1]. Hypomorphic alleles of the *age-1* [2] and *daf-2* [3] genes, orthologs of phosphoinositol-3-kinase [4], and the insulin/IGF-1 receptor [5] extend lifespan in *C. elegans* [6], [7]. Mutations in the insulin/IGF receptor (InR) also increase the median lifespan of female *Drosophila* [8] as do mutations in CHICO, the *Drosophila* IRS1 (insulin receptor substrate 1) ortholog [9]. Selman et al. [10] reported that female mice null for insulin receptor substrate 1 (Irs1) showed a 32% increase in median lifespan compared to WT while male *Irs1^−/−^* mice showed no significant increase in lifespan. In contrast, mice null for Irs2 die before 30 months of age. Taguchi et al. [11] reported that *Irs2^+/−^* mice lived 17% longer than WT mice. However, neither the number of mice nor the sex of the mice was given, and in a subsequent lifespan study, Selman et al. [12] found no significant increase in the lifespan of either male or female *Irs2^+/−^* mice compared to their WT littermates. Irs1 is thought to be more important in mitogenic signaling whereas Irs2 is more involved in metabolic signaling [13] so the more robust lifespan extending effect in Irs1 mutants could be due to reduced cell division while Irs2 mutants may fail to show robust lifespan extension due to metabolic dysregulation These data point to the complexities that alterations in components of the IGF-1/insulin signaling pathway might have on mammalian aging.

The most direct evidence that mutations affecting the insulin/IGF-1 signaling pathway lead to increased longevity in mammals has come from studies with *Igf1r^+/−^* mice (i.e., mice lacking one copy of the gene coding for IGF-1 receptor; mice lacking both copies die shortly after birth but the *Igf1r^+/−^* mice were reported to be phenotypically normal [14]). In 2003, Holzenberger et al. [15] reported that female *Igf1r^+/−^* mice exhibited a 33% increase in lifespan and were resistant to the oxidative stressor, paraquat. Males showed a statistically non-significant 16% increase in lifespan, and were not resistant to paraquat. These data supported the previous studies in invertebrates showing that reduced IGF-1 receptor (IGF-1R) signaling also leads to increased lifespan in mammals.

However, the lifespan data in the Holzenberger study are problematic because of the small sample size and the very short lifespan of both the wild type (WT) and *Igf1r^+/−^* mice studied (reviewed in [16] and [17]); therefore, we have reassessed the effect of reduced expression of the IGF-1R on lifespan using the rigorous criteria recommended by Ladiges et al. [18], e.g., lifespan and end-of-life pathology were assessed using large sample sizes and husbandry conditions that permitted the control lifespan to approach its full potential, which are necessary if the longevity differences in the experimental group are to be relevant to healthy aging. In agreement with Holzenberger et al. [15], we found that the female *Igf1r^+/−^* mice were more resistant to the oxidative stress than were WT female mice while no difference was observed between the male *Igf1r^+/−^* and WT mice. However, there was only a modest increase in the mean lifespan (4.7%) of female *Igf1r^+/−^* mice compared to their WT littermates and no significant change in end-of-life pathology. Thus, our data show that haploinsufficiency of *Igf1r* does not produce a robust increase in lifespan as previously reported, demonstrating that reduced IGF-1R signaling in mammals does not play the same major role in aging that is observed in invertebrates.

## Materials and Methods

### Ethics Statement

All procedures involving mice were approved by the subcommittee for Animal Studies at the Audie L. Murphy Veterans Administration Hospital (protocol #0508-001, “Role of IGF-1 Receptor in Aging and Age-Related Diseases”) and the University of Texas Health Science Center at San Antonio IACUC (protocol #06053, “IGF-1 Signaling and Aging”).

### Animals

The *Igf1r^+/−^* mice were kindly provided by Dr. Argiris Efstradiatis (Columbia University College of Physicians and Surgeons, New York) who derived them in a 129Sv background by homologous recombination, which ablated the third exon of the *Igf1r* gene [14]. The mice were backcrossed into the C57Bl/6 background for at least 10 generations. To establish the colony of mice used in this study, *Igf1r^+/−^* males were bred to C57BL/6 WT (i.e., *Igf1r^+/+^*) females purchased from the Jackson Laboratory (Bar Harbor, ME), producing offspring of which half were *Igf1r^+/−^* and half, WT. Mice were weaned into their final cages, genotyped, and randomly assigned to the experiments. In all studies described below, age-matched littermates were used as controls. The genotypes of the mice were determined as described by Liu et al. [14]. In the lifespan study, mice were maintained under pathogen-free barrier conditions and permitted to die of natural causes, i.e., there was no censoring of the animals. Cages assigned to longevity experiments were checked twice daily for dead animals but otherwise undisturbed. Upon death, the mice were necropsied for gross pathological lesions as previously described [19]. For each mouse, a list of pathological lesions was constructed and graded as previously described [19]–[22]. All mice were fed a standard NIH-31 chow *ad libitum* and maintained in micro-isolator cages, 4 to a cage, on a 12-hour dark/light cycle. C57Bl/6 mice are mature both sexually and with respect to body size at 6 months of age. At 25 months of age, typically fewer than 15% of the mice have died and pathology is minimum, so measurements are not confounded by disease/pathology. Therefore these were the two ages used in all the experiments presented here unless otherwise indicated.

### Paraquat and diquat

Paraquat was injected interperitoneally at dose of 50 mg/kg of animal body weight. Diquat was injected interperitoneally at a dose of 100 mg/kg for survival studies and 50 mg/kg for hepatotoxicity studies. A Hamilton syringe demarcated in 2.5 µl increments was used for the injection, making it possible to adjust dosage for body weight differences as small as 0.6 g. To track the survival of mice after paraquat or diquat treatment, the cages containing the treated mice were placed under an array of digital surveillance cameras (Strategic Vista, Ontario, Canada). These cameras monitored the animals continuously, and the footage was used to determine the time of death with a precision of 1 min. The time of injection was subtracted from the recorded time of death to obtain the survival time for each animal. For studies of diquat-induced hepatotoxicity, mice were anaesthetized with a standard ketamine/acepromazine/xylazine cocktail (0.02 cc/25 g body weight) 6 hr after diquat administration (50 mg/kg) and 200–400 µl of blood were collected by cardiac puncture then transferred to heparinized storage tubes. The animals were euthanized by cervical dislocation, and livers were removed and preserved in 10% neutral buffered formalin. Blood collected from diquat-treated mice was stored on ice in lithium heparin tubes, and as soon as possible after collection, plasma was separated by centrifugation for 10 min at 1.5 kG and 4°C. ALT activity in the plasma was measured as per manufacturer's instructions using the ALT Colorimetric Kit from Teco Diagnostics (Anaheim, CA). Liver samples in 10% neutral buffered formalin were paraffin embedded, sectioned, and fixed on slides. Apoptotic cells in these sections were identified on the basis of double strand DNA breaks using the ApopTag Kit from Chemicon (Temecula, CA, USA). Cell nuclei that were both dark and compacted were identified under a light microscope and scored as apoptotic. The number of apoptotic nuclei in the entire cross section was divided by cross sectional area (arbitrary grid units) to give number of apoptotic cells per cross sectional area of liver.

### Real-time PCR

Mice were fasted overnight and tissues were removed and frozen in liquid nitrogen. Total RNA was isolated using RNA STAT-60 (Tel-test, Friendswood, TX, USA). Single stranded cDNA was synthesized from 3.0 µg of RNA using the High-capacity cDNA Archive Kit (P/N 4322171; ABI, Foster City, CA, USA). Real-time PCR reaction was performed using TaqMan Universal PCR Master Mix (P/N 4324018) and TaqMan-MGB probes for IGF-1 (Mm00439561_m1), IGF-1R (Mm00802831_m1) and B2M (Mm00437762_m1) all of which were purchased from ABI. All samples were run in duplicate and quantitated in an ABI 7500 thermal cycler.

### *In vivo* IGF-1R signaling

After an overnight fast, mice were given an i.p. injection of 1 mg/kg body weight of rhIGF-1 (Austral Biologicals, San Ramon, CA, USA) or an equivalent volume of sterile saline. Ten min post injection, quadriceps muscle was collected and frozen in liquid nitrogen. Whole tissue homogenates were prepared and protein concentration was determined by Bradford assay [23]. For immunoblotting, primary antibody directed against IGF-1R (anti-IGF1R) and HRP-linked secondary antibody were purchased from Santa Cruz Biotechnology (Santa Cruz, CA, USA) and primary antibody directed against phospho-Akt (anti-pAkt Ser473) and total Akt (anti-Akt) were purchased from Cell Signaling Technologies (Danvers, MA, USA). For the glucose tolerance assay, mice were fasted overnight. Blood glucose was measured using an Accucheck glucometer (Roche Diagnostics, Indianapolis, IN, USA) at 0, 30, 60 and 120 min after i.p. injection of 2 g/kg body weight of dextrose. Insulin tolerance tests were performed by fasting the mice for 4 hr followed by i.p. injection of 0.5 U/kg body weight of insulin (Novolin, Novo Nordisk, Princeton, NJ, USA). Blood glucose was measured at 0, 30, 60 and 90 min. Serum IGF-1 assays of unstimulated WT and *Igf1r^+/−^* animals were performed using kits from ALPCO (Windham, NH, USA) as described by Delahunty et al. [24] to determine baseline circulating IGF-1 levelsanimals to determine circulating IGF-1 levels were performed using kits from ALPCO (Windham, NH, USA) as described by Delahunty et al. [24].

### Euglycemic hyperinsulinemic clamps

Insulin clamps were done as described by Wang et al. [25]. Five days prior to insulin clamp, mice were anesthetized using 150 mg ketamine, 30 mg xylazine and 5 mg acepromazine sc, and a catheter was inserted into the right jugular vein. Clamps were performed on awake, unrestrained mice. At the start of the experiment, a primed (5.3 ml/h×1 min) continuous (0.3 ml/h) infusion of human insulin (3.6 mU/min/kg) was started simultaneously with a variable infusion of 10% dextrose in order to maintain euglycemic conditions. Blood glucose levels were monitored by tail vein sampling. At the end of the clamp, animals are anesthetized with ketamine, and tissues were collected.

### Statistics

The Cox-Mantel Log rank test was used to evaluate all survival curves. Quantile regression as described by Koenker [26] was used to compare means and medians. The Student's t-test was used for all two-sample comparisons reported other than survival data. For all statistical tests returning significant p-values, these p-values are reported to the first significant digit. Pathological lesions in post-mortem samples collected from mice in the longevity study were graded for severity on a scale of 1–4. For each lesion in each organ, both severity and incidence were analyzed using the following formula:
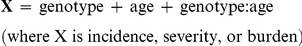
For incidence (presence or absence of a lesion) the above formula was used in a general linear model with a binomial error distribution. For severity, the above formula was used in a proportional-odds logistic regression model. For each organ, neoplastic, non-neoplastic, and overall disease burdens (i.e., the number of distinct lesions observed in that organ) were also compared as was the aggregate severity of each type of lesion (i.e., the number of different organs in which that lesion was observed). Finally, the animal-level disease burden (number of distinct lesions per animal) was compared for neoplastic, non-neoplastic, and overall lesions. For the three animal-level comparisons, the following lesions were only counted once per animal even if they were detected in more than one tissue: lymphacytic infiltration, lymphoma, suppurative inflammation, carcinoma, metastatic carcinoma, adenobranchiolar carcinoma, pheochromocytoma, and lymphoid hyperplasia. These tests were done for any lesion or grouping of lesions that occurred in more than five animals of the same genotype. Male and female data were analyzed separately. Altogether, 50 distinct lesions or groups of lesions were analyzed in males and 53 in females and the Holm [27] method was used to correct for multiple comparisons. The R statistical language [28] with the binom [29] and car [30] packages was used for the pathology analysis. In addition, the quantreg [26], surv2sample [31], and eha [32] packages were used for survival analysis.

## Results

### Characterization of *Igf1r^+/−^* Mice

**Figure 1:**
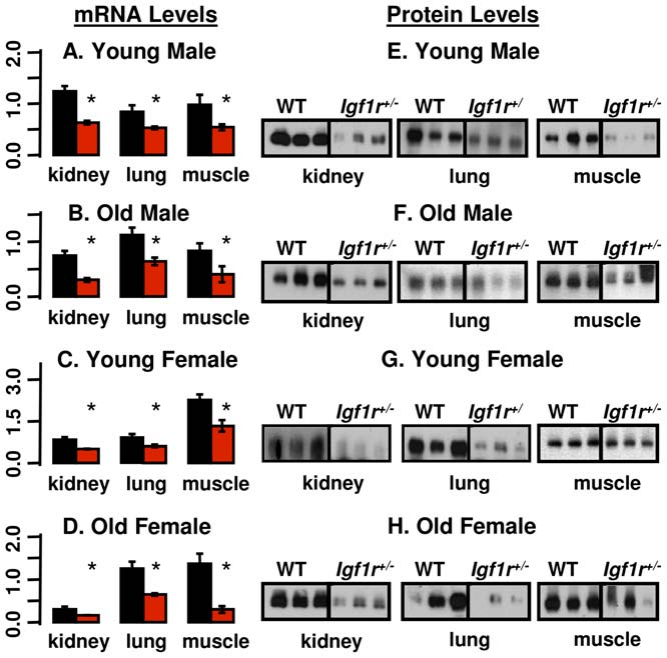
Igf1rβ expression. The mRNA and protein levels of the β subunit of Igf1r were measured in the kidney, lung, and muscle (quadriceps) from male and female mice that were 6 (Graphs A, E, C, and G) and 25 (Graphs B, F, D, and H) months old. The graphs on the left represent data from qRT-PCR and those on the right represent data from Western blots. Three to 6 animals were used per group. Black bars represent WT mice and red bars represent *Igf1r^+/−^* mice; the mean and SEM are shown, and asterisks indicate tissues showing a difference between WT and *Igf1r^+/−^* where p<0.05. The Student's t-test was used for the comparisons.

The body weights of male and female *Igf1r^+/−^* mice were 9% (20.8 vs. 22.8, p<0.001) and 12% (16.2 vs. 18.2, p<0.001) lower than those of their WT littermates, respectively. The expression of IGF-1R was measured in young (6 months) and old (25 months) WT and *Igf1r^+/−^* mice. The levels of *Igf1r* mRNA were significantly reduced (∼50%) in all tissues studied from young (Figures 1a and 1c) and old (Figures 1b and 1d) *Igf1r^+/−^* mice of both sexes and paralleled by a decrease in Igf1r protein levels as shown in Figures 1e and 1g which is similar to that reported for young mice by Holzenberger et al. [15]. However, we also show in Figures 1f and 1h that the expression of IGF-1R is reduced in old mice. The reduced expression of Igf1r had no effect on serum IGF-1 levels (Table S1) or tissue expression (mRNA levels) of IGF-1 in various tissues, with the exception of kidneys in young males (Table S2).

**Figure 2:**
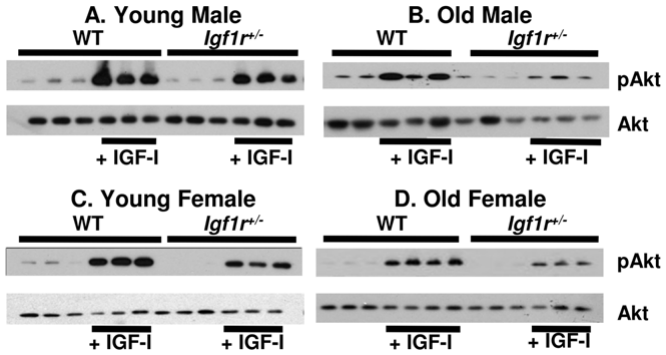
Induction of AKT phosphorylation by IGF-1 in WT and *Igf-1r^+/−^* mice. Levels of phosphorylated AKT were measured in the muscle (quadriceps) of 6- (graphs A and C) and 25- (graphs B and D) month-old male and female mice following injection of saline or rhIGF-1 (1 mg/kg body wt.) using Western blots as described in Materials and Methods. Three to 4 animals were used per group.

**Figure 3:**
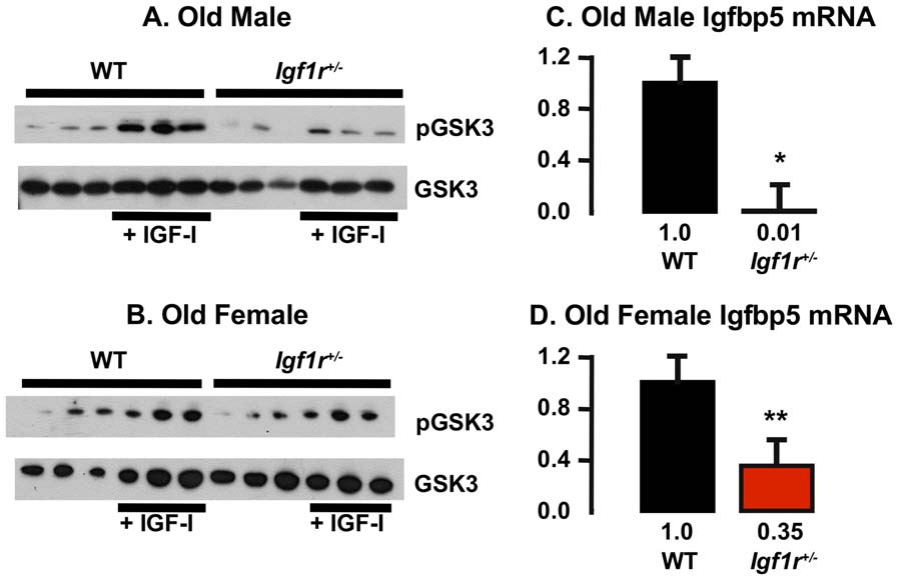
Induction of GSK3β phosphorylation and levels of Igfbp5 mRNA transcript in WT and *Igf1r^+/−^* mice. Levels of phosphorylated GSK3β were measured in the muscle (quadriceps) of 25-month-old male (graph A) and female (graph B) mice following injection of saline or rhIGF-1 (1 mg/kg body wt.) using Western blots as described in Materials and Methods. Three animals were used per group. The expression of Igfbp5 was measured in the same samples using qRT-PCR (graphs C and D). The vertical axis represents expression levels relative to B2M and the error bars represent SEM. P-values of 0.01 and 0.005 are represented by * and **, respectively.

Previously, Holzenberger et al. [15] showed that the IGF-1 signal as measured by IGF-1-induced phosphorylation of IGF-1R, IRS-1 and that Shc, was reduced in embryonic fibroblasts isolated from *Igf1r^+/−^* mice. To determine whether reduced IGF-1R levels compromise signaling in whole animals, we compared the ability of *Igf1r^+/−^* and WT mice to respond to a bolus of IGF-1 *in vivo* by measuring the phosphorylation of Akt in muscle. Akt phosphorylation in muscle was induced by IGF-1 in all mice: young (Figures 2a and 2c) and old (Figures 2b and 2d), WT and *Igf1r^+/−^*. However, induction of Akt in both male and female *Igf1r^+/−^* mice was approximately half that observed in the WT mice. Akt inhibits GSK3β by phosphorylating it, and IGF-1 signaling induces Igfbp5 expression [33], [34] therefore we also measured the phosphorylation of GSK3β and mRNA transcript levels of Igfbp5 in the quadriceps of WT and *Igf1r^+/−^* mice. We observed a decrease both in IGF-1 stimulated phosphorylation of GSK3bβ (Figures 3a and 3b) and in Igfbp5 transcript levels (Figures 3c and 3d), confirming a reduction of IGF-1 dependent signaling in *Igf1r^+/−^* mice as would be predicted for a biologically significant impairment of IGF-1R function.

### Glucose and Insulin Tolerance Tests

**Figure 4:**
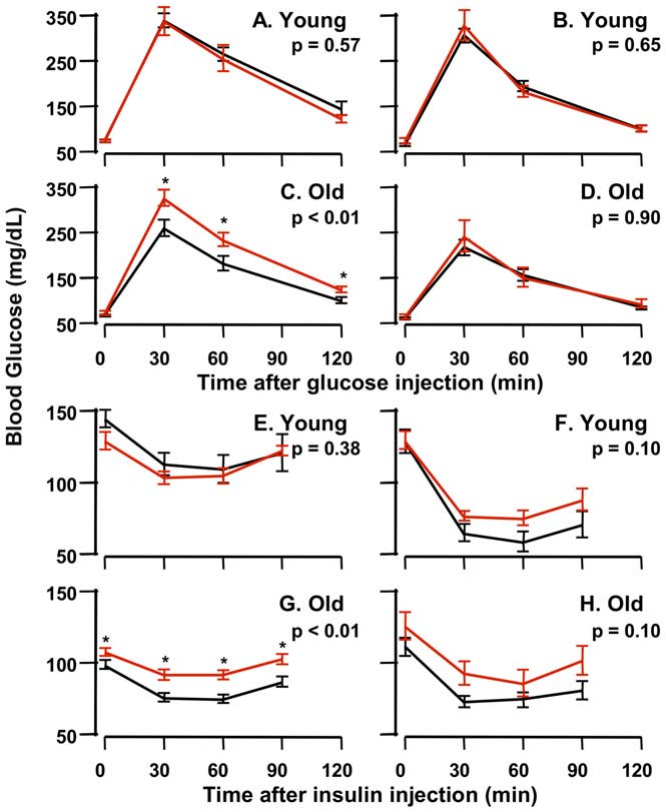
Glucose and Insulin Tolerance Tests. GTTs (2 g/kg, i.p.) were performed in 6-month-old male (A) and female (B) as well as 25-month-old male (C) and female (D) mice after a 12 hr fast, and blood glucose was recorded at times indicated. ITTs (0.5 U/kg, i.p.) were performed in 6-month-old male (E) and female (F) as well as 25-month-old male (G) and female (H) mice after a 5 hr fast. The data were obtained from 6 to 8 animals per group and the SEM is shown. Black lines show the blood glucose levels of WT (black lines) and *Igf1r^+/−^* mice (red lines). The Student's t-test was used for the comparisons of the areas under the curve (AUC) and the p-values are shown on each graph. Individual points were also compared in the same manner and corrected for multiple comparisons, and corrected p-values less than 0.05 are denoted by asterisks.

**Figure 5:**
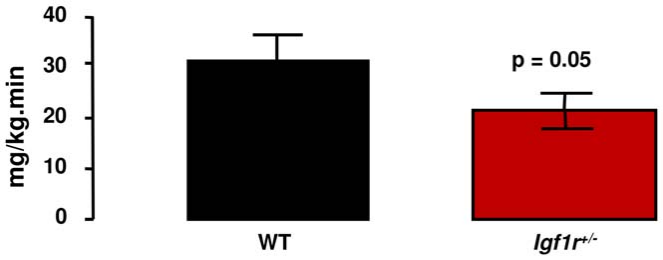
Peripheral Insulin Sensitivity. Peripheral (muscle) insulin sensitivity was measured with a 90 min hyperinsulinemic euglycemic clamp performed in 4 WT and 5 *Igf1r^+/−^* females, all 25 months old. Bars represent the average ± SE glucose infusion rate during the last 20 min of the clamp. A student's t-test was used to compare the infusion rates between the two groups, averaged over the 20 min period for each animal.

Holtzenberger et al. [15] reported that adult male *Igf1r^+/−^* mice, but not female *Igf1r^+/−^* mice, were less glucose tolerant than WT mice when given a bolus of glucose. In our study, we measured both glucose and insulin tolerance in young (5 months) and old (25 months) male and female *Igf1r^+/−^* and WT mice. We found that young male and female *Igf1r^+/−^* mice showed the same glucose tolerance as age-matched WT mice (Figures 4a and 4b). In contrast, old male *Igf1r^+/−^* mice were significantly less glucose tolerant compared to old male WT mice whether assessed by comparison of blood glucose levels at each time point post-glucose injection or as AUC (Figure 4c) while old female *Igf1r^+/−^*and WT mice showed no difference in glucose tolerance (Figure 4d). Of interest, when comparing young and old wt mice, old mice were more glucose tolerant than young mice and also had lower fed levels of serum glucose. Our observation of paradoxical age-related enhancement of glucose tolerance is exactly as has been reported previously in wild type C57BL/6J mice by Leiter's lab [35]. Young male and female *Igf1r^+/−^* and WT mice experienced similar declines in blood glucose after insulin injection (Figures 4e and 4f). )ld *Igf1r^+/−^* males showed a significantly attenuated response to an insulin challenge as compared to WT mice (Figure 4g). The old *Igf1r^+/−^* female mice, though glucose tolerant, displayed an overall trend toward insulin resistance compared to WT mice (Figure 4h). Therefore, we measured insulin sensitivity in the old female *Igf1r^+/−^* and WT mice using the hyperinslinemic-euglycemic clamp. As shown in Figure 5, the glucose infusion rate required to maintain euglycemia was significantly lower in the aged *Igf1r^+/−^* females as compared to the WT, indicating that female *Igf1r^+/−^* mice were less sensitive to the glucose lowering effect of insulin.

### Sensitivity of *Igf1r^+/−^* Mice to Oxidative Stress

**Figure 6:**
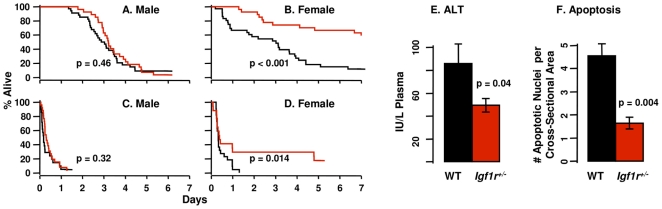
Sensitivity of WT and *Igf1r^+/−^* mice to oxidative stress. Paraquat (50 mg/kg) was administered to 37 male WT mice and 26 male *Igf1r^+/−^* mice (graph A) as well as to 39 female WT mice and 24 female *Igf1r^+/−^*mice (graph B). Diquat (50 mg/kg) was administered to 21 WT and 26 *Igf1r^+/−^* male mice (graph C) as well as to 22 WT and 17 *Igf1r^+/−^* female mice (graph D). The mice in graphs A–D were 5 to 9.5 months of age. Censored data points (due to uncertainty about the exact minute of an animal's death) are indicated by vertical tick-marks. Graphs E and F: Female WT and *Igf1r^+/−^* mice (10 to 11 months of age) were treated with diquat (50 mg/kg). Six hours after treatment the mice were killed and the ALT activities in the plasma (graph E) and number of apoptotic cells per unit area in a liver cross section (graph F) of 7 WT and 8 *Igf1r^+/−^*mice were determined. The mean and SEM are shown in the bar graphs. Black represents WT data and red represents *Igf1r^+/−^*data. Survival data were analyzed using the log-rank test while the ALT and apoptosis data were analyzed using Student's t-test and the p-values are shown.

Paraquat is a superoxide-anion generator that is commonly used to induce oxidative stress in cells and whole animals and was used by Holzenberger et al. [15] to show that female, but not male *Igf1r^+/−^* mice were more resistant to oxidative stress than WT mice. Male *Igf1r^+/−^* and WT mice show no statistically significant difference in survival when given a lethal dose of paraquat; 89% of the WT mice and 92% of the *Igf1r^+/−^* mice died within eight days (Figure 6a). However, female *Igf1r^+/−^* mice were more resistant to paraquat toxicity; 82% of the WT female mice died during the 8-day observation period compared to 37% of the *Igf1r^+/−^* mice (Figure 6b).

Because the effect of paraquat is concentrated mainly in the lung, we also compared the sensitivity of *Igf1r^+/−^* and WT mice to diquat, another superoxide anion generator that affects a variety of tissues to assess the generality of the increased resistance of the female *Igf1r^+/−^* mice to oxidative stress. Again, there was no significant difference between WT and *Igf1r^+/−^* male mice; all mice died within 31 hours of diquat administration (Figure 6c). However, the female *Igf1r^+/−^* mice showed a significantly increased resistance to diquat toxicity; all WT females died within 36 hours of treatment but only 60% of the *Igf1r^+/−^* females did so (Figure 6d). We also determined the sensitivity of the liver of female WT and *Igf1r^+/−^* mice to oxidative stress by measuring the activity of alanine-leucine transaminase (ALT) in the plasma six hours after treatment with a sub-lethal dose of diquat. ALT activity of *Igf1r^+/−^* mice was significantly (42%) lower compared to WT mice (Figure 6e) indicating reduced liver damage in the *Igf1r^+/−^* mice. In the same mice, we measured the induction of apoptosis in liver and found a significant reduction (64%) of apoptotic cells in *Igf1r^+/−^* mice compared to WT mice (Figure 6f). Thus, female *Igf1r^+/−^* mice show increased resistance to diquat-induced toxicity both in the liver and at the whole animal level.

### Longevity and End-of-Life Pathology of *Igf1r^+/−^* Mice

**Figure 7:**
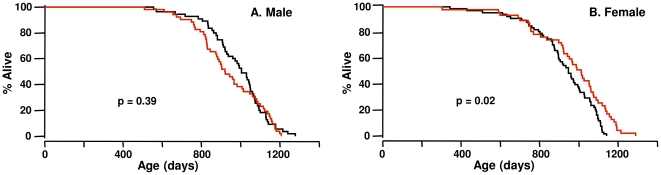
Longevity of WT and *Igf1r^+/−^* mice. The survival curves of 55 WT and 52 *Igf1r^+/−^* male mice (A) and 68 WT and 47 *Igf1r^+/−^* female (B) mice are shown in black for WT mice and red for *Igf1r^+/−^*mice. The survival curves were compared using the log-rank test, and the p-values are shown.

The survival curves for male and female *Igf1r^+/−^* and WT mice are shown in Figures 7a and 7b, respectively, and the detailed statistical analysis of the survival data are given in the Table 1. The mean survival of the WT male and female mice was 32.8 and 30.8 months, respectively, and fewer than 7% of the mice of either sex or genotype died before 20 months of age. Thus, the baseline lifespans in our aging colony are optimal, clearly not limited by disease or environmental stress and are representative of age-related processes independent of preventable extrinsic causes. The mean and median survival of the male *Igf1r^+/−^* mice were slightly (4 to 8%) shorter than the male WT mice; however, these differences were not statistically significant, nor were the overall distributions of survival times according the log-rank test. The female *Igf1r^+/−^* mice showed a 5 to 7% increase in mean, median, and 90^th^ percentile survival compared to female WT mice; and, these differences also were not significant; however, the overall distributions of survival times were significantly different at the P  =  0.02 level according to the log-rank test.

**Table 1:** A summary of lifespan data for WT and *Igf1r^+/^* ^−^ mice.

	Male				Female			
	WT	*Igf1r* ^+/−^	% Change	P	WT	*Igf1r* ^+/−^	% Change	P
N	55	52			68	47		
**Mean** **S EM**	98321	93924	−4.5	0.16	92321	96729	4.7	0.22
**Median** **(±95% CI)**	1004(937, 1048)	920(873, 1013)	−8.4	0.53	948(896,1001)	1013(924,1062)	6.9	0.64
**90^th^ Percentile** **(±95% CI)**	1139(1128, 1240)	1160(1138, 1205)	1.8	0.82	1110(1091, 1127)	1184(1143,1290)	6.7	0.26

In addition to the log-rank test, the means from the survival data shown in Figure 6A were compared using a bootstrap t-test and the medians and 90^th^ percentiles were compared using quantile regression as described in Materials and Methods.

We also conducted a comprehensive analysis of end-of-life pathology in the 222 mice used in the lifespan study. As expected for mice in the C57BL/6 background at the end of life [36], [37], the most common neoplastic lesion in both sexes and genotypes was lymphoma, which affected 75 to 89 percent of the mice. In male mice, the only lesions showing any evidence of a difference in incidence were the 36% decrease in all fatal tumors and the 47% decrease in fatal lymphoma in *Igf1r^+/−^* mice compared to WT mice (Table S3); however, these differences do not attain significance when corrected for multiple comparisons. In female mice, only the incidence of lymphocytic infiltrates, which is one measure of chronic inflammation, showed any evidence for a difference between *Igf1r^+/−^* and WT mice (a 30% decrease in *Igf1r^+/−^* mice) (Table S4); however, these differences also do not attain significance when corrected for multiple comparisons. We also measured the severity of the major pathological lesions in *Igf1r^+/−^* and WT mice (Table S5). The only lesions that showed any evidence for a difference in severity were all fatal tumors in males (a 36% decrease in *Igf1r^+/−^* mice) and lymphocytic infiltrates in females (a 52% decrease in *Igf1r^+/−^* mice). However again, these differences do not attain significance when corrected for multiple comparisons. The total disease burden and tumor burden of the *Igf1r^+/−^* and WT mice were also measured because previous studies show that disease burden is significantly reduced in established mouse models of longevity, e.g., dietary restriction [21], Ames Dwarf mice [20], and growth hormone receptor knockout mice [22]. No significant differences were observed in either disease or tumor burden between *Igf1r^+/−^* and WT mice for either males or females (Table S6). Thus, our detailed pathological analyses of *Igf1r^+/−^* mice show that reduced IGF-1 signaling had no major effect on end-of-life pathology in either male or female mice as would be predicted if reduced IGF-1 signaling delayed aging.

## Discussion

The major observation of our study is that reduced IGF-1 signaling had no significant effect on the mean, median, or 90% survival of either male or female *Igf1r^+/−^* mice compared to WT mice. Only when the overall distributions of survival times were analyzed by the log-rank test was a significant difference observed between WT and female *Igf1r^+/−^* mice (mean survival was increased less than 5%, p = 0.02). This finding is in sharp contrast to the previous study by Holzenberger et al. [15], which reported that the mean lifespan of female *Igf1r^+/−^* mice was increased 33% compared to WT mice (756±46 vs. 568±49 compared to the 967±29 vs. 923±21 from our study shown in Table 1). It should be noted that we studied larger cohorts of 47 to 68 animals, which allowed us to detect a 10% change in mean survival with a power of 0.8 [16].

We identified three likely explanations for the disparity between our observations on lifespan and those by Holzenberger et. al [15]. First, is the possibility that the mutations in the two *Igf1r^+/−^* mouse models are not equivalent because they were produced independently by two laboratories using different methods for generating knockout mice. The Holzenberger *Igf1r^+/−^* mice were produced by replacing exon 3 of *Igf1r* (which encodes most of the ligand binding domain on the α subunit of the receptor), with a loxP flanked exon 3 that had an adjacent neomycin resistance cassette. The entire segment was then deleted by crossing into Cre expressing mice. In contrast, the Efstratiadis group [14] performed a traditional knockout of the same exon, directly replacing it by homologous recombination. However, in both cases, virtually the same region was removed. Furthermore, both the Holzenberger laboratory [15], the Efstratiadis laboratory [14], and our laboratory observed ∼50% decrease in IGF1r expression in tissues of the *Igf1r^+/−^* mice using quantitative RT-PCR and Western blots. Therefore, we do not believe that the differences in how the respective knockout mice were generated have any effect of IGF-1R expression and are responsible for the contradictory lifespan data.

The second possibility is that the difference in lifespan is due to the genetic background of the *Igf1r^+/−^* mice because the mice used by Holzenberger et al. [15] were on the 129/J background, and the mice used in our study were on the C57Bl/6 background. We believe that it is unlikely that this is the reason for the contradictory lifespan data because all the other major phenotypes reported for the *Igf1r^+/−^* mice by Holzenberger et al. [15] are similar to what we observed. For example, both studies show the *Igf1r^+/−^* mice have slightly smaller body weights (∼10%) than WT mice, *Igf1r^+/−^* mice show ∼50% decrease in IGF-1R expression and reduced IGF-1 signaling, and male *Igf1r^+/−^* mice show impaired glucose tolerance on the glucose tolerance test. In addition, our data on the sensitivity of the mice to paraquat were virtually identical to that reported by Holzenberger et al. [15]; male *Igf1r^+/−^* and WT mice had a similar paraquat sensitivity while female *Igf1r^+/−^* mice showed significant resistance to paraquat compared to female WT mice. In an ongoing study, we are measuring the lifespan of female *Igf1r^+/−^* mice on a C57BL/6× 129Sv F1 background. As shown in Figure S1, with the majority of animals in both groups dead, we observe no statistically significant difference between the censored survival curves of WT and *Igf1r^+/−^* mice nor between median survival times. Thus, these preliminary data argue against strain background accounting for the observed results.

The final possibility, which we consider the most likely explanation for the differences between our lifespan data and those reported by Holzenberger et al. [15] is the number of animals used in the lifespan studies and the housing conditions. Holzenberger et al. [15] used a relatively small number of mice in their lifespan study (12 to 20 mice per group). In our study, 47 to 68 mice per group were used. Using larger sample sizes reduces the effects of uncontrolled variables, such as maternal- or paternal-specific effects on lifespan [38]. In other words, a larger sample size reduces the influence that each animal has on group summaries of survival. Therefore, the lifespan data are less likely to be distorted by any outlying observations and are more reproducible. However, we believe that differences in housing conditions, as evidenced by the length of the lifespans of the mice in the two studies, is also a major factor in the contradictory lifespan data. The mean lifespans of the WT and *Igf1r^+/−^* female mice reported by Holzenberger et al. [15] were 568 and 756 days, respectively, while the WT and *Igf1r^+/−^* male mice lived an average of 585 and 679 days, respectively. In fact, 40% of the female WT mice died by 12 months of age. In contrast, the mean lifespans of 129/J mice maintained at The Jackson Laboratory are reported to be 776 days and 855 days for female and male mice, respectively [39]. In other words, the lifespan of the WT 129/J female mice in the study by Holzenberger et al. [15] are 37% shorter than the lifespan reported at The Jackson Laboratory, and the *Igf1r^+/−^* female mice merely attain a normal mean lifespan for this strain of mice. The lifespan parameters of the C57BL/6 mice in our study are in line with or greater than that reported by other groups, e.g., National Institute on Aging [40] or The Jackson Laboratory [39], for C57Bl/6 mice maintained under contemporary pathogen-free, barrier conditions. For example, the mean lifespan of the male and female WT mice were 983 and 923 days, respectively, and fewer than 7% of the mice of either sex or genotype died before 20 months of age. By maximizing the lifespan of the mice, we have minimized the effect of genotype/environment interactions on lifespan, i.e., one has a more accurate measure of the effect of the genetic manipulation on aging. We propose that that the increase in the lifespan of the female and not male *Igf1r^+/−^* mice observed by Holzenberger et al. [15] was due largely to the increased resistance of the female *Igf1r^+/−^* mice to stress. Therefore, when the female mice are maintained in a more optimal and less stressful environment where they are able to live out their lifespan, such as in our study, no major difference in the lifespan of female *Igf1r^+/−^* and WT mice is observed. A similar observation was made for mice lacking methionine sulfoxide reductase-A (MsrA). Moskovitz et al. [41] reported that the MsrA knockout mice, which are sensitive to oxidative stress, had a shorter lifespan than WT mice when maintained in a colony with a relatively short lifespan (e.g., the mean lifespan of WT mice was 680 days). However, when the MsrA knockout mice were maintained under husbandry conditions that give optimal lifespan (e.g., the mean lifespan of the WT mice was 925 days), Salmon et al. [42] showed that the lifespan of the MsrA knockout and WT mice were identical.

Although lifespan data are critical in determining whether a manipulation retards aging, pathological data are also necessary because a pathological assessment gives one the likely cause of death and how the progression of pathological lesions are affected by the experimental manipulation tested, i.e., how broadly a manipulation affects age-related diseases [43]. For example, a wide variety of age-related pathological lesions are significantly delayed and/or reduced in three mouse models that show delayed aging, e.g., dietary restriction [21], [44], Ames Dwarf mice [20], and growth hormone receptor knockout mice [22]. In addition, these manipulations reduce the severity of many of the major pathological lesions and reduce the disease burden. The extensive pathological data we have obtained on the *Igf1r^+/−^* mice show no significant decrease in either the incidence or severity of any pathological lesion and no difference in disease burden compared to WT mice. Thus, the absence of an effect of IGF-1 signaling on end-of-life pathology is quite different than that observed with other manipulations that have been well documented to enhance longevity and retard aging in mice.

In summary, our lifespan and pathology data show that the *Igf1r^+/−^* mouse is not a model of delayed aging. These data have important consequences for the field of aging because it is well established that loss-of-function mutations in the insulin/IGF-1 signaling pathway lead to increased lifespan and an anti-aging phenotype in invertebrates [45]. Especially relevant to this study are the mutations in the *daf-2* gene in *C. elegans* and the InR gene in *Drosophila* that effect the function of the insulin/IGF-1 receptor. *C. elegans* and *Drosophila* have only one receptor for insulin and IGF-1, while mammals, such as mice and humans, have two different receptors, one for insulin and one IGF-1, each coded by a separate gene. Our data demonstrate quite clearly that reducing IGF-1 signaling approximately 50% in *Igf1r^+/−^* mice has very little effect on lifespan and end-of-life pathology, i.e., there is no evidence that these animals exhibit an anti-aging phenotype as has been observed in invertebrates when a loss of function occurs in the insulin/IGF-1 receptor. In addition, it has been argued that the decrease in circulating IGF-1 levels in dietary restricted mice [46], Ames and Snell dwarf mice [47], [48], and growth receptor knockout mice [49] plays an important role in the extended lifespan of these models through reduced IGF-1 signaling [45]. The *Igf1r^+/−^* mouse model has allowed us to study the effect of just reduced IGF-1 signaling on lifespan/aging from the many other pathways that are affected by dietary restriction and dwarfism. Our data demonstrate clearly that neither male nor female *Igf1r^+/−^* mice show the increase in lifespan or reduced/delayed pathology that is observed in male and female mice that are dietary restricted or have mutations resulting in the dwarf phenotype. Based on these data, we conclude that the reduced circulating IGF-1 levels in dietary restricted and dwarf mice play little if any role the anti-aging phenotype observed in these mice. In the case of dwarfs, it seems reasonable to conclude that reduced GH signaling per se is far more important than reduced IGF-1 in lifespan extension. Moreover, one common characteristic of dwarf mice and CR rodents is increased insulin sensitivity [50], [51]; in contrast, *Igf1r^+/−^* mice do not have increased insulin sensitivity, and in fact become insulin resistant as they age. In addition, old male *Igf1r^+/−^* mice develop glucose intolerance (with females exhibiting a tendency to glucose intolerance). Of interest with respect to gluco-regulation in mice, our old wt mice were more glucose tolerant than young wild type mice. This paradoxical enhancement of glucose tolerance in old C57Bl/6J mice has been previously reported by Leiter and colleagues [35], and has been attributed to increased beta cell insulin secretory capacity in old C57Bbl/6J wild type mice. This in turn suggests that the relative glucose-intolerance in old male *Igf1r^+/−^* mice is due to insulin resistance and a relative failure of the glucose sensing capacity in their beta cells thereby impairing glucose simulated insulin secretion. A direction for future research would be to test this hypothesis via islet morphometry and glucose-stimulated insulin release studies. Our present results suggest that haploinsufficiency of IGF-1R increases the probability for developing type 2 diabetes by diminishing peripheral insulin action and by preventing glucose stimulated compensatory increase in insulin secretion.

Our experiments follow the generally accepted reductionist paradigm in that optimal conditions (e.g., nutrition, temperature, humidity) and essentially a pathogen-free environment are used to isolate the underlying process of organismal aging from preventable pathologies as much as possible. In light of these results it appears that Holzenberger's group has demonstrated that female *Igf1r^+/−^* are rescued from some as-yet unidentified environmental stressor, and we have demonstrated that the intrinsic aging process is not affected by this mutation. However, as with any other animal experiment conducted under carefully controlled conditions, caution is advised in drawing inferences about other species, particularly humans. Even with access to state of the art health care, humans clearly do not live under optimal conditions. The stressor against which female *Igf1r^+/−^* mice are protected may well be clinically relevant and identifying this stressor would be an important avenue for future research. Such a search would nevertheless need as its starting point an environment where nothing interferes with an animal living to its naturally attainable lifespan *except* a candidate stressor against which the *Igf1r^+/−^* genotype is believed to be protective.

## Supporting Information

Figure S1 **Lifespan of Female WT and** ***Igf1r^+/−^*** **Mice on a C57BL/6X129Sv F1 Background.** Male C57Bl/6 *Igf1r^+/−^* mice were crossed to female 129 mice to generate female WT and *Igf1r^+/−^* mice on a C57BL/6x129Sv F1 background. Of the 66 WT mice, 47 mice died from natural causes, 5 mice were censored, and 14 mice were alive at the time of data analysis. Of 24 *Igf1r^+/−^* mice, 16 mice died of natural causes and 8 mice were alive at the time of data analysis. Lifespans of female C57BL/6× 129Sv F1 hybrids were analyzed using the log-rank test an no significant difference was found (P = 0.48). The median survivals were 1009 days (95% confidence interval 963–1079 days) for the WT mice and 1016 days (95% confidence interval 956–1170) for the *Igf1r^+/−^* mice.
(PDF)

Table S1 **Circulating Levels of IGF-1.** Serum from three mice from each group was assayed for IGF-1 as described in Materials and Methods, and the IGF-1 levels (expressed in ng/ml) are shown.
(PDF)

Table S2 **Igf1r Expression.** The mRNA levels of Igf1r were measured in the indicated tissues from male and female mice at 6 and 25 months of age. The mean and SEM columns are for ΔΔCT values of Igf1r mRNA normalized to the median expression level of the male WT group in each respective tissue. The Student's t-test was used for the comparisons. The p-values are shown with the tissue having a p<0.05, highlighted.
(PDF)

Table S3 **Incidence of Lesions in Males.** The presence or absence of each lesion or category of lesions shown was coded as 0 or 1, respectively, for each animal. Where a lesion was judged to have caused the death of an animal, the total incidence and the incidence of just the fatal instance of that lesion are shown on separate lines indented below the name of the lesion. Where data were obtained for both a category of lesion and organ-specific lesions within that category, the latter are shown indented below the name of the category. Otherwise the lesions are listed from most prevalent to least. Sample sizes vary because some tissues could not be analyzed due to autolysis. Lesions that had an incidence of 9 or more in the WT and/or Igf1r+/− mice were selected for statistical analysis. For each such lesion, a logistic regression model was fitted with incidence as the response variable and genotype, age, and the age-genotype as the covariates. The p-values from the genotype and age:genotype effects were adjusted for multiple comparisons using the Holm method [27]. The raw and adjusted p-values for the genotype effect are shown. None of the age:genotype p-values approached significance. The highlighted rows indicate lesions where the uncorrected p-values are less than 0.05. Given that none of these values were significant after adjustment, the highlighted values should be interpreted as a possibly meaningful trend rather than a significant difference.
(PDF)

Table S4 **Incidence of Lesions in Females.** See legend for table S3.
(PDF)

Table S5 **Males and Females, Severity.** Organ-specific lesions (glomerulonephritis, gonadal degeneration, nephrocalcinosis, pituitary adenoma, and subscapular hyperplasia) were assigned a severity grade as described in Methods by Ikeno et. al. [20]–[22]. For lymphoma and lymphocytic infiltration, the number of organs where those lesions were observed was used as a measure of whole-organism severity. A logistic regression model was fitted to the organ-specific data and a linear model was fitted to the lymphoma and lymphocytic infiltrate data. In all cases, severity was the response variable and genotype, age and the age-genotype interaction were the covariates. The p-values for the genotype and age-genotype effects were adjusted for multiple comparisons using the Holm method [27]. The raw and adjusted p-values for the genotype effect are shown. None of the age-genotype p-values approached significance. The highlighted rows indicate lesions where the uncorrected p-values are less than 0.05. Given that none of these values were significant after adjustment for multiple comparisons, the highlighted values should be interpreted as a possibly meaningful trend rather than a strongly significant difference.
(PDF)

Table S6 **Males and Females, Disease Burden.** Disease burden is defined as the number of distinct lesions observed in an animal (either total lesions or only neoplastic ones) as described in Materials and Methods. In calculating neoplastic burden, lesions were counted separately for each organ in which they were observed with the exception of lymphoma, which was counted only once regardless of how many organs it was found in. The p-values from the genotype and genotype-age effects were adjusted for multiple comparisons using the Holm method [27]. The raw and adjusted p-values for the genotype effect are shown. None of the genotype-age p-values approached significance.
(PDF)
