# Supplementary material for: Does Reduced IGF-1R Signaling in Igf1r +/− Mice Alter Aging?
Source: PLoS One. 2011 Nov 23;6(11):e26891. doi: 10.1371/journal.pone.0026891 (PMC3223158; doi:10.1371/journal.pone.0026891)
Supplement: Figure S1 — Lifespan of Female WT and Igf1r+/− Mice on a C57BL/6X129Sv F1 Background. Male C57Bl/6 Igf1r+/− mice were crossed to female 129 mice to generate female WT and Igf1r+/− mice on a C57BL/6x129Sv F1 background. Of the 66 WT mice, 47 mice died from natural causes, 5 mice were censored, and 14 mice were alive at the time of data analysis. Of 24 Igf1r+/− mice, 16 mice died of natural causes and 8 mice were alive at the time of data analysis. Lifespans of female C57BL/6× 129Sv F1 hybrids were analyzed using the log-rank test an no significant difference was found (P = 0.48). The median survivals were 1009 days (95% confidence interval 963–1079 days) for the WT mice and 1016 days (95% confidence interval 956–1170) for the Igf1r+/− mice. (PDF) [file pone.0026891.s001.pdf]

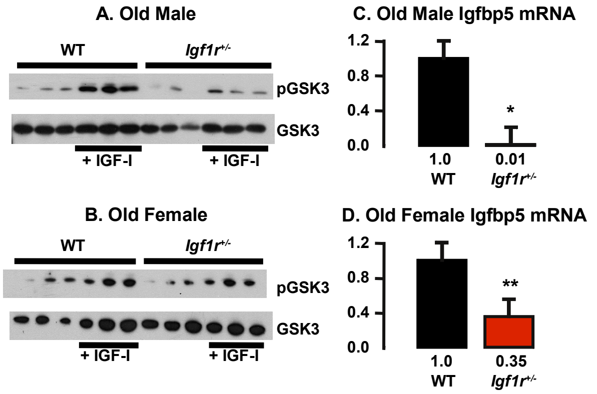

Figure S1. Induction of GSK3β phosphorylation and levels of Igfbp5 mRNA transcript in WT and *Igf1r*$^{+/-}$ mice. Levels of phosphorylated GSK3β were measured in the muscle (quadriceps) of 25-month-old male (Graph A) and female (Graph B) mice following injection of saline or rhIGF-1 (1 mg/kg body wt.) using Western blots as described in Materials and Methods. Three animals were used per group. The expression of Igfbp5 was measured in the same samples using qRT-PCR (Graphs C and D). The vertical axis represents expression levels relative to B2M and the error bars represent SEM. P-values of 0.01 and 0.005 are represented by * and **, respectively.

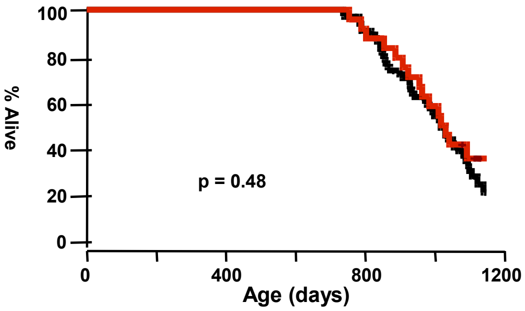

Figure S2. Lifespan of Female WT and *Igf1r*$^{+/-}$ Mice on a C57BL/6X129Sv F1 Background. Male C57Bl/6 *Igf1r*$^{+/-}$ mice were crossed to female 129 mice to generate female WT and *Igf1r*$^{+/-}$ mice on a C57BL/6x129Sv F1 background. Of the 66 WT mice, 47 mice died from natural causes, 5 mice were censored, and 14 mice were alive at the time of data analysis. Of 24 *Igf1r*$^{+/-}$ mice, 16 mice died of natural causes and 8 mice were alive at the time of data analysis. Lifespans of female C57BL/6 x 129Sv F1 hybrids were analyzed using the log-rank test an no significant difference was found (P=0.48). The median survivals were 1009 days (95% confidence interval 963-1079 days) for the WT mice and 1016 days (95% confidence interval 956-1170) for the *Igf1r*$^{+/-}$ mice.
