# Supplementary material for: Does Reduced IGF-1R Signaling in Igf1r +/− Mice Alter Aging?
Source: PLoS One. 2011 Nov 23;6(11):e26891. doi: 10.1371/journal.pone.0026891 (PMC3223158; doi:10.1371/journal.pone.0026891)
Supplement: Table S1 — Circulating Levels of IGF-1. Serum from three mice from each group was assayed for IGF-1 as described in Materials and Methods, and the IGF-1 levels (expressed in ng/ml) are shown. (PDF) [file pone.0026891.s002.pdf]

Table S1. Circulating Levels of IGF-1

6 Months Old

	Mean	SEM	P
**Male WT**	230	24	0.82
**Male *Igf1r*$^{+/-}$**	236	7	
**Female WT**	183	15	0.12
**Female *Igf1r*$^{+/-}$**	246	29	

25 Months Old

	Mean	SEM	P
**Male WT**	252	15	0.56
**Male *Igf1r*$^{+/-}$**	263	11	
**Female WT**	232	11	0.33
**Female *Igf1r*$^{+/-}$**	247	10	
