# Supplementary material for: Does Reduced IGF-1R Signaling in Igf1r +/− Mice Alter Aging?
Source: PLoS One. 2011 Nov 23;6(11):e26891. doi: 10.1371/journal.pone.0026891 (PMC3223158; doi:10.1371/journal.pone.0026891)
Supplement: Table S2 — Igf1r Expression. The mRNA levels of Igf1r were measured in the indicated tissues from male and female mice at 6 and 25 months of age. The mean and SEM columns are for ΔΔCT values of Igf1r mRNA normalized to the median expression level of the male WT group in each respective tissue. The Student's t-test was used for the comparisons. The p-values are shown with the tissue having a p<0.05, highlighted. (PDF) [file pone.0026891.s003.pdf]

Table S2. Igf1r Expression

Sex	Age	Tissue	WT			Igf1r+/-		
			N	Mean	SEM	N	Mean	SEM
Male	6 Months	Kidney	3	1.04	0.06	3	0.66	0.08
		Lung	4	1.35	0.26	4	0.89	0.10
		Muscle	4	0.87	0.16	4	1.02	0.15
	25 Months	Kidney	4	0.94	0.17	4	1.10	0.25
		Lung	6	0.78	0.10	6	0.89	0.05
		Muscle	5	0.69	0.09	4	0.44	0.10
Female	6 Months	Kidney	3	0.91	0.11	3	0.87	0.20
		Lung	4	0.94	0.18	4	1.07	0.16
		Muscle	4	0.53	0.06	4	0.91	0.27
	25 Months	Kidney	3	0.87	0.05	3	0.82	0.04
		Lung	6	0.84	0.05	5	0.72	0.06
		Muscle	3	0.45	0.09	3	0.26	0.03
