# Supplementary material for: Does Reduced IGF-1R Signaling in Igf1r +/− Mice Alter Aging?
Source: PLoS One. 2011 Nov 23;6(11):e26891. doi: 10.1371/journal.pone.0026891 (PMC3223158; doi:10.1371/journal.pone.0026891)
Supplement: Table S3 — Incidence of Lesions in Males. The presence or absence of each lesion or category of lesions shown was coded as 0 or 1, respectively, for each animal. Where a lesion was judged to have caused the death of an animal, the total incidence and the incidence of just the fatal instance of that lesion are shown on separate lines indented below the name of the lesion. Where data were obtained for both a category of lesion and organ-specific lesions within that category, the latter are shown indented below the name of the category. Otherwise the lesions are listed from most prevalent to least. Sample sizes vary because some tissues could not be analyzed due to autolysis. Lesions that had an incidence of 9 or more in the WT and/or Igf1r+/− mice were selected for statistical analysis. For each such lesion, a logistic regression model was fitted with incidence as the response variable and genotype, age, and the age-genotype as the covariates. The p-values from the genotype and age:genotype effects were adjusted for multiple comparisons using the Holm method [27]. The raw and adjusted p-values for the genotype effect are shown. None of the age:genotype p-values approached significance. The highlighted rows indicate lesions where the uncorrected p-values are less than 0.05. Given that none of these values were significant after adjustment, the highlighted values should be interpreted as a possibly meaningful trend rather than a significant difference. (PDF) [file pone.0026891.s004.pdf]

Table S3. Incidence of Lesions in Males

	WT			Igf1r$^{+/-}$				
	N	Positive		N	Positive			
		#	%		#	%	Raw P	Adjusted P
**Neoplastic Lesions**								
Total	49	40	81.6	59	46	78.0	0.84	1.00
Fatal	49	28	57.1	59	21	35.6	0.03	0.61
**Lymphoma**								
Total	49	37	75.5	59	38	64.4	0.77	1.00
Fatal	49	25	51.0	59	16	27.1	0.02	0.41
**Adenoma, Lung**								
Total	48	4	8.3	57	7	12.3	-	-
Fatal	48	2	4.2	57	4	7.0	-	-
**Adenoma, Thyroid**								
Total	38	1	2.6	41	2	4.9	-	-
Fatal	38	1	2.6	41	1	2.4	-	-
**Non-Neoplastic Lesions**								
Total	49	46	93.9	59	53	89.8	0.57	1.00
Fatal	49	3	6.1	59	2	3.4	-	-
**Glomerulonephritis**								
Total	49	24	49.0	59	37	62.7	0.11	1.00
Fatal	49	2	4.1	59	1	1.7	-	-
**Degeneration, Gonad**	48	22	45.8	59	29	49.2	0.45	1.00
**Lymphocytic Infiltrate**	49	26	53.1	59	25	42.4	0.20	1.00
**Kidney**	49	14	28.6	59	14	23.7	0.58	1.00
**Lung**	48	8	16.7	57	10	17.5	0.91	1.00
**Salivary Gland**	45	5	11.1	56	4	7.1	-	-
**Liver**	49	2	4.1	59	5	8.5	-	-
**Intestine**	49	3	6.1	58	1	1.7	-	-
**Other Reproductive Organs**	49	2	4.1	59	1	1.7	-	-
**Subcapsular Hyperplasia, Adrenal Gland**	47	12	25.5	59	17	28.8	0.45	1.00
**Nephrocalcinosis**	49	8	16.3	59	10	16.9	0.48	1.00
**Suppurative Inflammation**	49	7	14.3	59	7	11.9	-	-
**Psammoma Bodies, Brain**	49	8	16.3	59	3	5.1	-	-
**Sperm Cell Granulosa**	49	3	6.1	59	2	3.4	-	-
**Other Reproductive Organs**	49	2	4.1	59	1	1.7	-	-
**Congestion & Edema, Lung**	48	1	2.1	57	4	7.0	-	-
**Ulcer, Stomach**	49	2	4.1	58	1	1.7	-	-
**Fatty Change, Liver**	49	0	0.0	59	3	5.1	-	-
**Angiectasia, Liver**	49	2	4.1	59	0	0.0	-	-
**Cortical Hyperplasia, Adrenal Gland**	47	2	4.3	59	0	0.0	-	-
**Exocrine Atrophy, Pancreas**	46	2	4.3	55	0	0.0	-	-
**Lymphoid Hyperplasia, Lymph Nodes**	47	0	0.0	58	2	3.4	-	-
