# Supplementary material for: Does Reduced IGF-1R Signaling in Igf1r +/− Mice Alter Aging?
Source: PLoS One. 2011 Nov 23;6(11):e26891. doi: 10.1371/journal.pone.0026891 (PMC3223158; doi:10.1371/journal.pone.0026891)
Supplement: Table S4 — Incidence of Lesions in Females. See legend for table S3. (PDF) [file pone.0026891.s005.pdf]

Table S4. Incidence of Lesions in Females

	WT			$Igf1r^{+/-}$				
	N	Positive		N	Positive			
		#	%		#	%	Raw P	Adjusted P
**Neoplastic Lesions**								
Total	57	48	84.2	46	42	91.3	0.59	1.00
Fatal	57	34	59.6	46	24	52.2	0.78	1.00
**Lymphoma**								
Total	57	43	75.4	46	41	89.1	0.15	1.00
Fatal	57	25	43.9	46	21	45.7	0.38	1.00
**Adenoma, Pituitary**								
Total	48	9	18.8	40	3	7.5	0.13	1.00
Fatal	48	6	12.5	40	3	7.5	-	-
**Adenoma, Thyroid**								
Total	37	5	13.5	38	3	7.9	-	-
Fatal	37	3	8.1	38	1	2.6	-	-
**Adenoma, Lung**								
Total	57	3	5.3	46	1	2.2	-	-
Fatal	57	2	3.5	46	0	0.0	-	-
**Hemangiosarcoma**	57	2	3.5	46	0	0.0	-	-
**Non-Neoplastic Lesions**								
Total	57	55	96.5	46	45	97.8	0.61	1.00
Fatal	57	9	15.8	46	6	13.0	0.60	1.00
**Glomerulonephritis**								
Total	57	45	78.9	46	39	84.8	0.20	1.00
Fatal	57	7	12.3	46	6	13.0	-	-
**Subcapsular Hyperplasia, Adrenal Gland**	57	39	68.4	46	39	84.8	0.17	1.00
**Lymphocytic Infiltrate**	57	32	56.1	46	18	39.1	0.05	1.00
**Kidney**	57	21	36.8	46	11	23.9	0.09	1.00
**Lung**	57	14	24.6	46	5	10.9	0.04	0.97
**Salivary Gland**	54	7	13.0	45	3	6.7	-	-
**Urinary**	57	4	7.0	45	2	4.4	-	-
**Intestine**	57	3	5.3	46	2	4.3	-	-
**Liver**	57	2	3.5	46	0	0.0	-	-
**Nephrocalcinosis**	57	11	19.3	46	15	32.6	0.51	1.00
**Congestion & Edema, Lung**	57	5	8.8	46	2	4.3	-	-
**Endometrial Hyperplasia**	57	4	7.0	46	3	6.5	-	-
**Centrolobular Necrosis, Liver**	57	3	5.3	46	1	2.2	-	-
**Erosion, Stomach**	57	1	1.8	46	3	6.5	-	-
**Follicular Hyperplasia, Thyroid**	37	2	5.4	38	0	0.0	-	-
**Follicular Hyperplasia, Thyroid**	37	2	5.4	38	0	0.0	-	-
**Interstitial Hyperplasia, Pancreas**	52	0	0.0	44	2	4.5	-	-
