# Supplementary material for: Does Reduced IGF-1R Signaling in Igf1r +/− Mice Alter Aging?
Source: PLoS One. 2011 Nov 23;6(11):e26891. doi: 10.1371/journal.pone.0026891 (PMC3223158; doi:10.1371/journal.pone.0026891)
Supplement: Table S5 — Males and Females, Severity. Organ-specific lesions (glomerulonephritis, gonadal degeneration, nephrocalcinosis, pituitary adenoma, and subscapular hyperplasia) were assigned a severity grade as described in Methods by Ikeno et. al. [20]–[22]. For lymphoma and lymphocytic infiltration, the number of organs where those lesions were observed was used as a measure of whole-organism severity. A logistic regression model was fitted to the organ-specific data and a linear model was fitted to the lymphoma and lymphocytic infiltrate data. In all cases, severity was the response variable and genotype, age and the age-genotype interaction were the covariates. The p-values for the genotype and age-genotype effects were adjusted for multiple comparisons using the Holm method [27]. The raw and adjusted p-values for the genotype effect are shown. None of the age-genotype p-values approached significance. The highlighted rows indicate lesions where the uncorrected p-values are less than 0.05. Given that none of these values were significant after adjustment for multiple comparisons, the highlighted values should be interpreted as a possibly meaningful trend rather than a strongly significant difference. (PDF) [file pone.0026891.s006.pdf]

# Table S5. Males and Females, Severity

| Males | WT | | | Igf1r$^{+/-}$ | | | | |
|---|---|---|---|---|---|---|---|---|
| | N | Mean | SEM | N | Mean | SEM | Raw P | Adjusted P |
| **Lymphoma** | | | | | | | | |
| Total | 49 | 2.15 | 0.26 | 59 | 1.80 | 0.24 | 0.71 | 1.00 |
| Fatal | 49 | 0.63 | 0.10 | 59 | 0.31 | 0.07 | 0.02 | 0.25 |
| **Lymphocytic Infiltrate** | 49 | 0.75 | 0.12 | 59 | 0.60 | 0.11 | 0.27 | 1.00 |
| **Glomerulonephritis** | 49 | 0.67 | 0.12 | 59 | 0.71 | 0.09 | 0.39 | 1.00 |
| **Degeneration, Gonad** | 48 | 0.88 | 0.16 | 59 | 0.90 | 0.14 | 0.63 | 1.00 |
| **Subcapsular Hyperplasia, Adrenal Gland** | 47 | 0.40 | 0.11 | 59 | 0.41 | 0.10 | 0.62 | 1.00 |
| **Nephrocalcinosis** | 49 | 0.31 | 0.11 | 59 | 0.24 | 0.08 | 0.61 | 1.00 |

| Females | WT | | | Igf1r$^{+/-}$ | | | | |
|---|---|---|---|---|---|---|---|---|
| | N | Mean | SEM | N | Mean | SEM | Raw P | Adjusted P |
| **Lymphoma** | | | | | | | | |
| Total | 57 | 2.88 | 0.36 | 46 | 3.46 | 0.32 | 0.36 | 1.00 |
| Fatal | 57 | 0.44 | 0.07 | 46 | 0.54 | 0.10 | 0.14 | 1.00 |
| **Lymphocytic Infiltrate** | 57 | 0.91 | 0.14 | 46 | 0.50 | 0.10 | 0.03 | 0.59 |
| **Glomerulonephritis** | 57 | 1.37 | 0.13 | 46 | 1.46 | 0.14 | 0.59 | 1.00 |
| **Subcapsular Hyperplasia, Adrenal Gland** | 57 | 1.54 | 0.18 | 46 | 1.87 | 0.16 | 0.19 | 1.00 |
| **Nephrocalcinosis** | 57 | 0.32 | 0.10 | 46 | 0.61 | 0.15 | 0.41 | 1.00 |
| **Adenoma, Pituitary** | 48 | 0.56 | 0.18 | 40 | 0.25 | 0.14 | 0.14 | 1.00 |
