# Supplementary material for: Does Reduced IGF-1R Signaling in Igf1r +/− Mice Alter Aging?
Source: PLoS One. 2011 Nov 23;6(11):e26891. doi: 10.1371/journal.pone.0026891 (PMC3223158; doi:10.1371/journal.pone.0026891)
Supplement: Table S6 — Males and Females, Disease Burden. Disease burden is defined as the number of distinct lesions observed in an animal (either total lesions or only neoplastic ones) as described in Materials and Methods. In calculating neoplastic burden, lesions were counted separately for each organ in which they were observed with the exception of lymphoma, which was counted only once regardless of how many organs it was found in. The p-values from the genotype and genotype-age effects were adjusted for multiple comparisons using the Holm method [27]. The raw and adjusted p-values for the genotype effect are shown. None of the genotype-age p-values approached significance. (PDF) [file pone.0026891.s007.pdf]

Table S6. Males and Females, Disease Burden

	WT			$Igf1r^{+/-}$				
	**N**	**Mean**	**SEM**	**N**	**Mean**	**SEM**	**Raw P**	**Adjusted P**
**Male, Total Disease Burden**	49	3.68	0.25	59	3.55	0.22	0.86	1.00
**Male, Neoplastic Disease Burden**	49	0.96	0.08	59	0.96	0.08	0.51	1.00
**Female, Total Disease Burden**	57	4.15	0.22	46	4.07	0.21	0.47	1.00
**Female, Neoplastic Disease Burden**	57	1.23	0.10	46	1.11	0.07	0.11	1.00
